# Multifaceted Roles of GSK-3 in Cancer and Autophagy-Related Diseases

**DOI:** 10.1155/2017/4629495

**Published:** 2017-12-12

**Authors:** Romina Mancinelli, Guido Carpino, Simonetta Petrungaro, Caterina Loredana Mammola, Luana Tomaipitinca, Antonio Filippini, Antonio Facchiano, Elio Ziparo, Claudia Giampietri

**Affiliations:** ^1^Department of Anatomical, Histological, Forensic Medicine and Orthopedic Sciences, Sapienza University of Rome, Rome, Italy; ^2^Department of Movement, Human and Health Sciences, Division of Health Sciences, University of Rome “Foro Italico”, Rome, Italy; ^3^Istituto Dermopatico dell'Immacolata Istituto di Ricovero e Cura a Carattere Scientifico (IDI-IRCCS, FLMM), Rome, Italy

## Abstract

GSK-3 is a ubiquitously expressed serine/threonine kinase existing as GSK-3*α* and GSK-3*β* isoforms, both active under basal conditions and inactivated upon phosphorylation by different upstream kinases. Initially discovered as a regulator of glycogen synthesis, GSK-3 is also involved in several signaling pathways controlling many different key functions. Here, we discuss recent advances regarding (i) GSK-3 structure, function, regulation, and involvement in several cancers, including hepatocarcinoma, cholangiocarcinoma, breast cancer, prostate cancer, leukemia, and melanoma (active GSK-3 has been shown to induce apoptosis in some cases or inhibit apoptosis in other cases and to induce cancer progression or inhibit tumor cell proliferation, suggesting that different GSK-3 modulators may address different specific targets); (ii) GSK-3 involvement in autophagy modulation, reviewing signaling pathways involved in neurodegenerative and liver diseases; (iii) GSK-3 role in oxidative stress and autophagic cell death, focusing on liver injury; (iv) GSK-3 as a possible therapeutic target of natural substances and synthetic inhibitors in many diseases; and (v) GSK-3 role as modulator of mammalian aging, related to metabolic alterations characterizing senescent cells and age-related diseases. Studies summarized here underline the GSK-3 multifaceted role and indicate such kinase as a molecular target in different pathologies, including diseases associated with autophagy dysregulation.

## 1. GSK-3 Structure and Regulation

GSK-3 is a serine/threonine kinase existing as two isoforms named GSK-3*α* (51 KDa) and GSK-3*β* (47 KDa), expressed in most tissues and encoded by two different genes. According to the bodymap analysis available at IST Online Medisapiens (http://ist.medisapiens.com/#bodymap), the expression is ubiquitous but shows relevant differences in different tissues. For instance, GSK-3*α* is much less expressed in the nerves, ovary, and skin, while it is expressed at higher levels in the reticulocytes, appendix, whole blood, and pituitary gland. On the other hand, GSK-3*β* is much less expressed in the reticulocytes, dura mater, lymph node, and pancreas, while it appears expressed at higher levels in the blood NK cells and bone marrow granulocytes.

**Figure 1:**
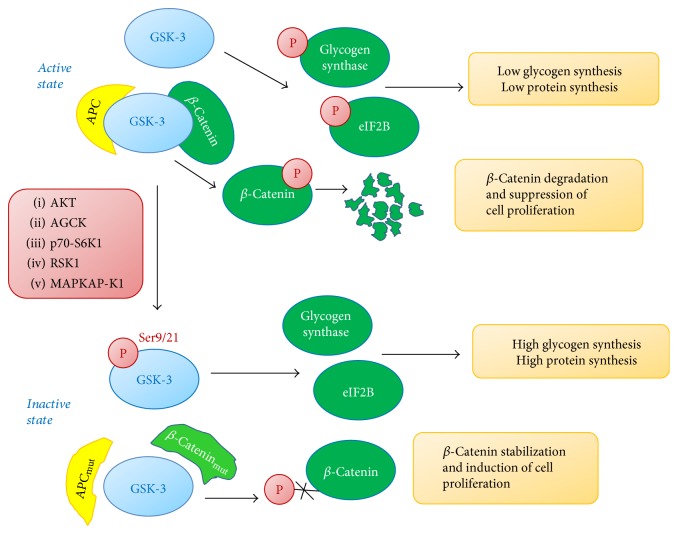
GSK-3 regulation.

An alternative splice variant of GSK-3*β*, named GSK-3*β*2, has also been reported [1]. Both isoforms are monomeric and comprise a highly conserved catalytic domain (about 98% identity). Such high rate of similarity explains why the two isoforms phosphorylate the same targets [2]. The GSK-3 three-dimensional structure resembles that of mitogen-activated protein kinase (MAPK) family members and the fully active conformation depends on its interaction with the substrate which previously undergone a “priming phosphorylation event” by other kinases [3]. The larger mass of GSK-3*α* compared to GSK-3*β* is due to its glycine-rich N-terminal tail, responsible for the GSK-3*α* cytoplasm localization, while GSK-3*β*, which lacks the glycine-rich domain, has a nuclear and cytoplasmatic localization [4, 5]. Other main differences fall in their C-termini, showing only 36% identity in the last 76 residues of the two isoforms. Under basal conditions, both proteins are active. GSK-3*β* constitutive activation seems to occur *via* phosphorylation in tyrosine 216 [6]. Phosphorylation in N-terminal serine 21 and serine 9, respectively, of GSK-3*α* and GSK-3*β* by AKT leads to their inactivation and consequently glycogen and protein synthesis increase. The serine residue on GSK-3 has been also shown to be phosphorylated by other kinases, such as AGC kinases, p70 ribosomal S6 kinase-1 (p70-S6 K1), p90 ribosomal S6 kinase (RSK1), and MAPK-activated protein kinase-1 (MAPKAP-K1, also known as RSK). In addition to its posttranslational regulation through phosphorylation, GSK-3 activity may be modulated through its association with other proteins. In particular, GSK-3 interaction with axin has been well studied and demonstrated to be crucial for GSK-3-dependent regulation of canonical WNT signaling pathway [6]. A schematic representation of GSK-3 inhibition through phosphorylation by different kinases is shown in Figure 1.

## 2. Signaling Pathways Regulated by GSK-3

GSK-3 was originally demonstrated to play an important role in regulating glycogen synthesis, as one of the molecular events involved in insulin signaling. Insulin activates phosphatidyl-inositide 3-kinase (PI3K) which in turn activates 3-phosphoinositide-dependent protein kinase 1 (PDK1), thus leading to AKT kinase phosphorylation. The latter phosphorylates and inhibits GSK-3, leading to dephosphorylation of GSK-3 substrates such as glycogen synthase and eukaryotic initiation factor 2B (eIF2B), finally promoting conversion of glycogen synthase to its active form and stimulating both glycogen and protein synthesis [3]. Amino acids have also been shown to inhibit GSK-3; this occurs *via* the mammalian target of rapamycin (mTOR) and the downstream S6K1 kinase [7]. Growth factors such as EGF may inhibit GSK-3 by both MAPK pathway and PI3-kinase/AKT pathway, and tumor-promoting phorbol esters can inhibit GSK-3 via MAPK cascade [8]. Furthermore, a WNT-induced inhibition of GSK-3 has been described. In the absence of WNTs, GSK-3 is active and phosphorylates axin, *β*-catenin, and adenomatous polyposis coli (APC). Under this condition, *β*-catenin undergoes ubiquitin-mediated proteolytic degradation. When WNTs bind their frizzled receptors, through the key transducer Dishevelled (DVL) phosphoprotein, stabilization and accumulation of *β*-catenin occur; this event is dependent on GSK-3 inhibition due to phosphorylation at a residue different from that targeted by AKT [3]. In fact, although AKT signaling leads to inhibition of GSK-3 via serine phosphorylation, AKT signaling does not cause stabilization and accumulation of *β*-catenin [9, 10]. It has also shown *β*-catenin accumulation in the presence of highly active GSK-3, and this is dependent on APC or *β*-catenin mutations [11, 12]. Given the role of active GSK-3 in promoting degradation of oncogenic proteins such as *β*-catenin, it may acquire tumor suppressor properties. Conversely, upon canonical WNT signaling, inactive GSK-3 fosters cell proliferation by *β*-catenin stabilization thus acquiring tumor-promoting activity (Figure 1). Since *β*-catenin is an essential component of cadherin-based adhesion junctions, GSK-3 also regulates cell adhesion via *β*-catenin accumulation. Interestingly, it has been also shown that WNT signaling does not directly inactivate GSK-3 but more likely disrupts the formation of the “*β*-catenin destruction complex” [13].

**Figure 2:**
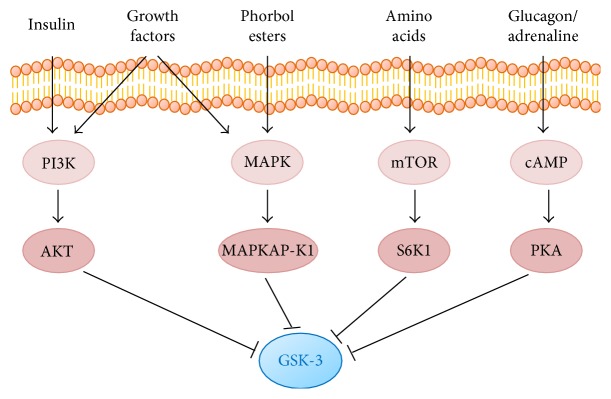
Signaling pathways leading to GSK-3 inactivation.

GSK-3 can be phosphorylated and inhibited by cyclic AMP- (cAMP-) dependent protein kinase/protein kinase A (PKA) in the presence of high cAMP levels, following glucagon or adrenaline stimulus. Remarkably, GSK-3 phosphorylation can also be achieved by incubation with cAMP-elevating agents or cAMP analogues [14]. A schematic representation of signaling pathways responsible for GSK-3 inhibition is shown in Figure 2. The inhibition of GSK-3 by the different pathways generally leads to dephosphorylation of its substrates. Phosphorylation of GSK-3 substrates generally leads to their inactivation, and many substrates require an additional “priming phosphorylation event” which is performed by a different kinase and occurs at a site located C-terminally to the site phosphorylated by GSK-3. Extensive lists of GSK-3 substrates or GSK-3 binding proteins have been reported and include amyloid precursor protein, APC, ATP-citrate lyase, axin, axil, *β*-catenin, c-jun, Jun B, Jun D, Ci155, C/EBP alpha, CRMP2, CRMP4, CREB, CTP, cyclin D1, dystrophin, eIF2B, glycogen synthase, glucocorticoid receptor, heat shock factor 1, hnRNP, K-casein, KRP, MAB 1B, MAP 2, MAP 2C, MITF, c-Myc, L-Myc, alpha NAC nascent polypeptide-associated complex, NCAM, NDRG1, NDRG2, neurofilament L, neurofilament M, neurofilament H, Notch 1C, p21 CIP1, p53, presenilin, pyruvate DH, PP1 G-subunit, protein phosphatase inhibitor 2, stathmin, synphilin-1, RSK1, and Tau (https://thebiogrid.org/ and http://www.genecards.org/).

GSK-3-dependent substrate phosphorylation may represent a signal toward their degradation. In fact, when GSK-3 phosphorylates cyclin D1 at threonine 286 and c-myc at threonine 58 they undergo ubiquitylation and proteolytic degradation. For such a reason, upon GSK-3 inhibition, growth factors may lead to both cyclin D1 and c-myc stabilization. Also, the transcription factor c-jun may be phosphorylated by GSK-3 and this event suppresses its DNA binding activity. Therefore, GSK-3 inhibition is able to enhance c-jun potential to stimulate the transcription of several genes including those involved in cell cycle progression [15].

## 3. Role of GSK-3 in Apoptosis

It is now clear that GSK-3 plays a pivotal role in numerous cellular functions, other than regulator of glycogen metabolism. As reported below, active GSK-3 has been shown to induce apoptosis in some cases and to inhibit apoptosis in other cases. Cooper and collaborators first demonstrated that, while GSK-3 overexpression induces apoptosis in different cell lines (i.e., pheochromocytoma PC12 cells and Rat-1 fibroblasts), overexpression of a GSK-3 inactive mutant prevents apoptosis [16]. Other studies performed using specific GSK-3 inhibitors confirm this finding [17]. We have previously addressed this issue in skeletal muscle tissue and demonstrated that decreased GSK-3*β* serine-9 phosphorylation leads to increased active caspase-3 and cytochrome *c* release [18]. GSK-3*β* has been shown to be directly involved in cell death mediated by PI3K/mTOR inhibitor and by pan-histone deacetylase (HDAC) inhibitor, in lymphoma cell lines [19]. Interestingly, trichostatin A, a histone deacetylase inhibitor (HDACI), induces apoptosis through GSK-3*β* in MCF-7 breast cancer cells [20], and a specific GSK-3 inhibitor (SB-415286) induces apoptosis in different leukemia cell lines [21]. In neurons, GSK-3*β* exerts a proapoptotic action inducing mitochondrial translocation of the proapoptotic Bcl-2 family member Bax, which occurs after GSK-3*β-*dependent phosphorylation of Bax in Ser163 [22]. Moreover, GSK-3*β* inhibition significantly reduces hepatic apoptotic cell death in response to D-galactosamine/LPS-induced liver injury [23] and improves the survival of mice with polymicrobial sepsis, ameliorating liver injury, with a mechanism involving its ability to inhibit inflammatory response by modulation of NF-𝜅B and CREB activation [24]. These data suggest that inhibition of GSK-3*β* may act as a relevant complementary strategy to the antibiotic treatment opening an interesting scenario in the development of novel antimicrobial strategies. As a further indication of the role of GSK-3 in apoptosis regulation, GSK-3*β* KO mouse has been reported to die in utero and this phenotype is likely dependent on an apoptosis defect [25]. As discussed in more details in the next section, GSK-3 may have a relevant effect on cancer cell apoptosis, likely via *β*-catenin. In fact, on one hand, it has been demonstrated that GSK-3 regulates axins, intracellular *β*-catenin antagonists, and cell fate regulators, while on the other hand inhibition of GSK-3 enhances TRAIL-induced apoptosis [26] as well as sorafenib-induced apoptosis in melanoma cells [27].

## 4. Opposite Role of GSK-3 in Cancer Progression/Setup

GSK-3 role in cancer progression is largely investigated and still debated. In fact, in some cases, GSK-3 activity has been associated with tumor progression, while in other cases suppression of GSK-3 activity by different kinases has been associated with cancer progression, for instance, by stabilizing components of the *β*-catenin complex. GSK-3*β* inhibition leads to *β*-catenin activation and tumor cell proliferation [28]. However, GSK-3 is overexpressed in various cancer conditions such as colon, liver, ovarian, and pancreatic tumors and GSK-3*β* downregulation inhibits pancreatic cancer growth, angiogenesis, and vascular endothelial growth factor expression [29–32]. GSK-3 role in cancer is often dependent on GSK-3-driven mammalian target of rapamycin (mTOR), a signaling molecule crucial in cell proliferation. mTOR is found in two complexes, mTOR complex-1 (mTORC1) and mTOR complex-2 (mTORC2). Signaling through mTORC1 is involved in tumor progression, and remarkably, GSK-3 inhibitors have been shown to inhibit mTORC1 activity [33]. Recently, GSK-3 involvement has been demonstrated in a study reporting that differentiation-inducing factor-1 displays a strong antimelanoma activity exerted in two ways, the first (i.e., antiproliferation action) involving a GSK-3-dependent degradation of cyclin D1 and c-Myc and the second (i.e., antimigration and anti-invasion) involving a GSK-3-independent mechanism [34]. Further, GSK-3 directly induces growth and survival in human melanoma cells, by increasing levels of the Pax3 transcription factor [35].

According to GEO database (https://www.ncbi.nlm.nih.gov/sites/GDSbrowser?acc=GDS1375), we observed that 63 samples of human melanoma and benign nevi reported in the dataset GDS 1375 [36] show GSK-3*α* expression significantly upregulated in melanoma biopsies as compared to benign nevi human biopsies (1200 units versus 901 units, *p* < 0.0001), while GSK-3*β* expression appears unmodified (791 units versus 680 units, *p* < 0.2). Such observation was confirmed by the additional data reported in IST Online Medisapiens dataset (http://ist.medisapiens.com) collected from the 355 samples of melanoma and healthy skin, and all together support the hypothesis of a differential role of GSK-3*α* and GSK-3*β* in melanoma biology.

*β*-Catenin regulation by GSK-3*β* has been shown to play a key role in hepatocellular carcinoma (HCC). An enhanced activation of WNT/*β*-catenin pathway is often found in several types of cancers; it may be considered an early event in hepatocarcinogenesis and correlates with an aggressive phenotype [37]. In addition, the liver carcinogenesis induced by HCV has been related to the HCV core protein ability to stabilize *β*-catenin by inhibiting GSK-3*β* [38]. Furthermore, in HCC, several molecular mechanisms involving genetic and epigenetic alterations have been shown [39]. One mechanism involves insulin and IGF-1. They inhibit GSK-3*β* [40], leading to nuclear localization of *β*-catenin [41] which binds its nuclear targets, such as TCF/LEF-1, and induces gene transactivation and tumor formation [42].

Usually, AKT is activated in human cancers, including carcinomas, glioblastoma multiforme, and various hematological malignancies. Noteworthy, while activated AKT inhibits GSK-3 through the phosphorylation of GSK-3 at Ser21/Ser9, however, such inactivation does not always affect *β*-catenin levels in the cell and does not completely inhibit GSK-3. For instance, two pancreatic cancer cell lines, PANC1 and ASPC1, exhibit amplification of AKT and high levels of AKT RNA and protein [43] but also highly active GSK-3*β* suggesting that, although some pools of GSK-3 can be phosphorylated by AKT at Ser21/Ser9 and inhibited, other pools of GSK-3 may remain active in cancer cells [31]. Moreover, another study has shown high levels of active AKT in human colorectal carcinomas, but levels of inactive phospho-GSK-3*β* Ser9 are lower than in their normal counterparts [30]. Altogether, these studies suggest that AKT activation and GSK-3 inhibitory phosphorylation are not always correlated *in vivo* in human tumors and part of GSK-3 remains active in cancer cells irrespective of AKT activation.

Data available indicate GSK-3*β* as a crucial gatekeeper to maintain a regular cell proliferation rate and conditions favorable to cell death activation. This suggests that the persistent inhibition of GSK-3*β* may favor oncogenic conditions. The autocrine stimulation of an IGF-1 R-dependent signaling pathway is one of these conditions. Moreover, GSK-3 interacts with other signaling pathways implicated in HCC pathogenesis, such as Notch, Hedgehog (HH), and TGF-*β* pathways. Many studies demonstrate the aberrant activation of HH [44] and Notch signaling [45]. In the latter, GSK-3 is an important component, although its role remains controversial. In fact, in some studies, GSK-3 activity has been reported to enhance nuclear localization and transcriptional activity by phosphorylation of two domains in Notch1 intracellular portion [46]. On the other hand, other studies report that GSK-3 phosphorylates and decreases Notch protein levels and downregulates its transcriptional activity [47]. Finally, the TGF-*β* pathway may have dichotomous function, with both pro- and antitumor activities. In fact, in early steps of hepatocarcinogenesis, TGF-*β* shows tumor-suppressive properties while in late stage, it promotes tumor progression by stimulating epithelial-mesenchymal transition (EMT), cell invasion, and cancer metastasis [48]. In hepatocytes, TGF-*β*, through a Src-dependent pathway, activates ERK5 that can phosphorylate GSK-3*β* on serine 9, inhibiting its activity [49]. TGF-*β*, by inhibiting GSK-3 kinase activity, interferes with phosphorylation of the tumor suppressor hepatocyte nuclear factor 4 alpha (HNF4*α*), a transcription factor controlling the expression of EMT master genes such as SNAI1; this results in its functional inactivation and contributes to EMT progression.

**Figure 3:**
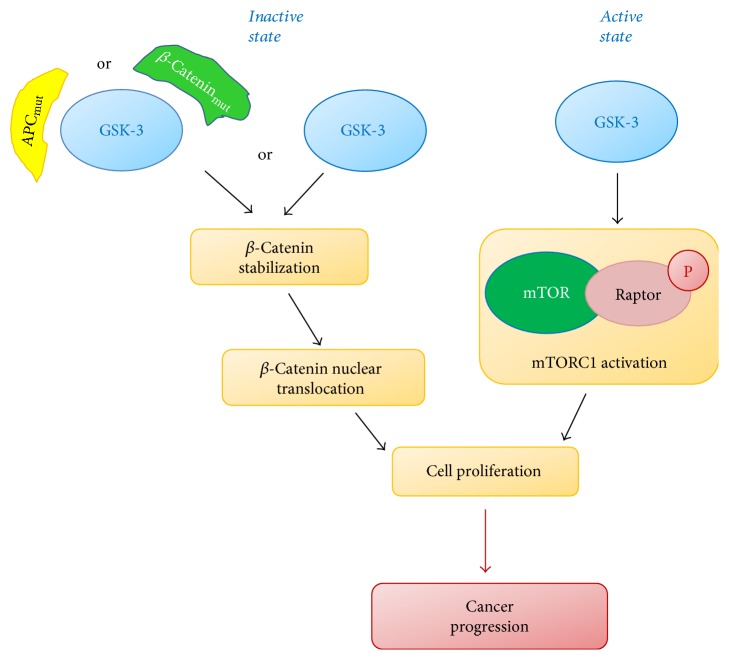
Opposite role of GSK-3 in cancer.

Cholangiocarcinoma (CCA) is the second most common primary hepatobiliary cancer that originates from biliary epithelium cells known as cholangiocytes [50, 51]. GSK-3*β* plays an important role in CCA, by mediating the cross-talk of PI3K/AKT and WNT/*β*-catenin pathways directly controlling cell growth in a cholangiocarcinoma setup [52]. Remarkably, GSK-3 *α*/*β* phosphorylation in serine 21/9 appears to be strongly increased in cholangiocarcinoma tissues as compared to normal biliary tissues and to be significantly associated with tumor progression. Also, P-glycoprotein (P-gp) is intrinsically overexpressed in many tumors, affecting the colon, rectum, pancreas, liver, kidneys, and bile ducts [53]. It is known to play a pivotal role in multidrug resistance (MDR), which reduces chemotherapy efficacy in CCA [54]. For that reason, several potent P-gp-dependent MDR reversers have been studied and the saponin mixture *β*-escin combined with other drugs such as 5-FU and VCR has shown remarkable inhibitory and synergic effects in CCA cells [55]. Interestingly, *β*-escin increases cholangiocarcinoma cells line sensitivity to chemotherapy, by inducing GSK-3*β* phosphorylation and dephosphorylation at tyrosine-216 and serine-9, respectively, leading to *β*-catenin degradation [55]. Finally, prostaglandin E2 (PGE2) is known to induce cholangiocarcinoma cell proliferation and invasion in a GSK-3-mediated way [56]. Altogether, all these studies reveal that, although its protumor or antitumor role is still debated depending on the cellular context, GSK-3 may be considered a promising molecular target in different tumors. A schematic representation illustrating the opposite models of GSK-3 involvement in cancer is shown in Figure 3. It suggests that different GSK-3 modulators (activators or inhibitors) should be further explored to address their specific effect in cancer treatment.

## 5. Role of GSK3 in Autophagy

**Figure 4:**
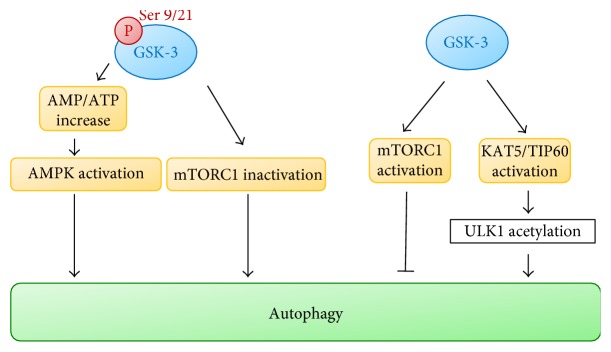
Models of GSK-3 involvement in autophagy.

Autophagy is a complex molecular mechanism involved in disassembling unnecessary or dysfunctional cellular components through double-membrane vesicles named autophagosomes, ultimately fusing with lysosomes, leading to their degradation through lysosome hydrolases. This process is usually activated under nutrient deprivation [57]. Autophagy starts with the formation of an isolation membrane called phagophore; then, the phagophore edges fuse to form a double-membrane vesicle, named autophagosome, sequestering the cytoplasmatic material to be eliminated. This process is performed through a complex molecular machinery including mTOR, which therefore represents a critical autophagy regulator [58, 59]. mTOR kinase is a sensor of intracellular amino acids, ATP, and hormones and acts as an autophagy inhibitor. It is inhibited by the autophagy inducer rapamycin, controls the autophagy onset, and is responsible for S6K and 4EBP1 phosphorylation [60]. Two ubiquitin-like conjugation pathways are involved in autophagosome formation, namely, the autophagy-related (ATG) 8 and ATG12 protein systems. Such two systems control phosphatidylethanolamine conjugation to mammalian LC3. As a result, the soluble LC3-I is converted to LC3-II, recruited to the autophagosomal membrane; therefore, such molecule is usually exploited to monitor autophagy [61]. Despite the large investigation regarding the role of autophagy in tumor formation and metabolism, its precise function is still debated since it has demonstrated both tumor-promoting and tumor-suppressing properties [62]. Autophagy, by releasing metabolic precursors necessary for macromolecular biosynthesis or ATP generation, makes energy available to tumor cells undergoing metabolic stress. On the other hand, autophagy genes are frequently monoallelically deleted, silenced, or mutated in different human tumors, thus supporting the autophagy tumor-suppressing properties [63]. Therefore, while during cancer initiation, autophagy may suppress tumor progression and autophagy deregulation may contribute to genomic instability; in the later stages, it may facilitate tumor progression supporting cancer cell survival, particularly in the presence of therapy-induced stress. GSK-3 role in autophagy regulation has been studied in the past few years. GSK-3 inhibits autophagy through the mammalian target of rapamycin (mTOR) complex 1 (mTORC1). In fact, overexpression of either GSK-3*α* or GSK-3*β* activates mTORC1 and suppresses autophagy in MCF-7 breast cancer cells. Conversely, treating cells with GSK-3 inhibitors inhibits mTORC1 activity and increases autophagic flux [33]. It has been clarified that GSK-3 regulates mTORC1 by phosphorylating the mTOR-associated scaffold protein raptor (regulatory-associated protein of mTOR) on Serine 859. GSK-3 inhibition reduces mTOR and raptor interaction leading to reduced phosphorylation of both p70S6K1 and ULK-1 and to increased autophagic flux [64]. In human breast cancer cells, GSK-3 overexpression increases the autophagosome number by autophagic flux inhibition. This activity has been directly related to reduced lysosomal acidification triggered by GSK-3 [33]. Furthermore, GSK-3 inhibition induces prosurvival autophagy in human pancreatic cancer cells. This occurs through GSK-3 dependent regulation of the transcription factor EB (TFEB), that is, a master regulator of autophagy and lysosomal biogenesis [65]. In a prostate cancer cell model, inhibition of GSK-3*β* activity leads to a significant increase of AMP/ATP ratio, a strong trigger of AMPK activation, thus leading to autophagy induction [66]. Inoki and colleagues have also shown that GSK-3 inhibits mTOR pathway by phosphorylating the tumor suppressor TSC2 in an AMPK-priming phosphorylation-dependent manner. Therefore, sequential phosphorylation of TSC2 by AMPK and GSK-3 occurs and these events may lead to mTOR pathway inhibition [67]. GSK-3 commonly accepted involvement in autophagy regulation is schematically represented in Figure 4.

Alterations of autophagic pathways have been extensively investigated in degenerative diseases and have been shown to be the central mechanisms in the pathogenesis of amyotrophic lateral sclerosis. Interestingly, a small heterocyclic GSK-3 inhibitor is able to induce the recovery of neurological symptoms in amyotrophic lateral sclerosis condition [68]. Autophagy impairment has been reported in other neurodegenerative processes. In fact, upon neurotoxin intoxication, astrocytes undergo autophagic flux block that can be rescued by rapamycin or by GSK-3*β* inhibition [69]. GSK3 overactivity has been reported to occur in sporadic Alzheimer's disease (AD) cases and therefore may play an important role in disease progression. GSK-3 mediates the hyperphosphorylation of tau (one of the brain microtubule-associated proteins), the increased production of *β*-amyloid from *β*-amyloid precursor protein (via *β* and *γ* secretase-mediated cleavage), and ultimately leads to autophagy impairment. More in detail, GSK-3*α*, but not GSK-3*β*, has been shown to regulate *β*-amyloid precursor protein cleavage resulting in the increased production of *β*-amyloid plaques. Since the discovery of its involvement in AD [70], GSK-3 has been proposed as a new target enzyme and is expected to provide a novel avenue for therapeutic intervention in AD. In Parkinson's disease (PD), the GSK3-*β* inhibitor lithium decreases the aggregation and phosphorylation of *α*-synuclein and leads to increased autophagy. Conversely, GSK3*β* activation depresses autophagy and increases the total protein level and phosphorylation of *α*-synuclein [71].

Data reported in literature indicate that autophagy is regulated by GSK-3 mostly via mTORC1. It has also been clarified that GSK-3, in the absence of growth factors, is able to activate the acetyltransferase KAT5/TIP60, which in turn stimulates the protein kinase ULK1 to induce autophagy [72]. Remarkably, GSK-3 seems to play a key role also in stemness; in fact, inhibition of both GSK-3 and mTORC1 induces a proautophagic gene signature in hematopoietic stem cells, which is crucial to maintain their self-renewal ability [73].

In the liver, several autophagy pathways have been identified and characterized [74]. Selective autophagy contributes to several physiological functions, representing a mechanism exploited by hepatocytes in order to modulate their metabolic capability [74]. Hepatic autophagy mostly depends on the fasting–feeding cycle and is under hormones and amino acid control [74]. Hepatic autophagy has a key role in the adaptation to starvation, inducing glycogenolysis, lipolysis, and protein catabolism. Furthermore, quality and quantity control of mitochondria and peroxisomes can directly regulate hepatic metabolism through *β*-oxidation [74]. In fasting, early-phase glucagon leads to GSK-3 inhibition and promotes hepatocyte glycogenolysis in order to maintain blood glucose levels [75]. Moreover, upon nutrient deprivation, hepatocytes upregulate the transcription of genes related to *β*-oxidation and autophagy, thus leading to lipophagy with subsequent *β*-oxidation and ketone body production [74, 76]. Of notice, differently from GSK-3*β* KO mice, GSK-3*α* KO mice are not embryonically lethal although they have metabolism defects such as enhanced glucose and insulin sensitivity [77] further supporting the involvement of GSK-3 in autophagy-dependent metabolic processes. Furthermore, it has been suggested that persistent phosphorylation of GSK-3*β* may have a fundamental impact on glycogen metabolism and cell growth in hepatoma cells [78].

Given its role in metabolic balance and organelle quality control, an unbalance or malfunction of autophagy pathways in hepatocytes has been associated with the pathogenesis of several liver diseases, including nonalcoholic fatty liver disease (NAFLD), alcoholic fatty liver (AFL), viral hepatitis, and liver cancer [79]. NAFLD is one of the most important causes of liver-related morbidity in obese children and adults [80–82]. Both NAFLD and AFL are characterized by hepatocyte steatosis. In NAFLD, fatty liver is mostly due to continuous dietary intake of excess dietary fat in the absence of excess alcohol consumption [79, 81, 82]. Differently, in AFL, steatosis is due to ethanol metabolism which leads to increased production of highly reactive acetaldehyde, fatty acid ethyl esters, and phosphatidylethanol [83]. Interestingly, both NAFLD and AFL are histologically characterized by impaired autophagy associated with prominent SQSTM1 protein accumulation in the form of cytoplasmic inclusions, histologically known as Mallory bodies [74, 84]. It has been suggested that inhibition of GSK-3*β* activity may be considered an important strategy to reverse the imbalanced oxidation and the impaired autophagy and ameliorate liver conditions [85].

Selective autophagy in hepatocytes may represent a defense mechanism against lipid accumulation [86]; however, lipotoxicity effects can prevail and suppress autophagic activity [87]. In fact, autophagy enhancement using pharmaceutical agents alleviates liver steatosis [88, 89] and contributes to Mallory body degradation [90]. NAFLD progression involves inflammation (nonalcoholic steatohepatitis (NASH)), fibrosis, and cirrhosis. In this context, the activation of hepatic stellate cells (HSCs) plays a key role in the progression toward fibrosis and cirrhosis [86]. Under normal conditions, HSCs are quiescent vitamin A­storing cells; however, in a diseased liver, HSCs are activated and change to myofibroblast-like cells. Activated HSCs acquire proliferative, contractile, and inflammatory properties and produce extracellular matrix compounds, thus resulting in fibrogenesis [91]. Interestingly, during this process, quiescent HSCs lose their lipid stores and autophagy may act by cleaving retinyl esters within cytoplasmic droplets [91]. It should be noted that selective knockout of autophagy-related genes in mouse HSCs inhibits experimental induced fibrogenesis [79, 92]. Thus, autophagy may support HSC activation resulting in enhanced fibrogenesis [91]. Therefore, although autophagy may have beneficial effect on hepatocyte steatosis in NAFLD, it may also induce HSC activation resulting in enhanced fibrogenesis [74].

Besides the role in hepatocytes and HSC, autophagy pathways are also investigated in the pathogenesis of biliary tree disorders [93]. Fibrosing cholangiopathies are a heterogeneous group of diseases affecting cholangiocytes (i.e., the parenchyma cells lining bile ducts) and comprising primary biliary cholangitis (PBC), primary sclerosing cholangitis, and biliary atresia [94]. Accumulation of LC3-positive vesicles and p62 aggregation were described in primary biliary cholangitis; autophagy deregulation may induce cholangiocyte senescence, which in turn is involved in the immune-mediated bile duct pathologies. Cholangiocytes can acquire a senescence-associated phenotype responsible for aberrant expression of chemokines, cytokines, and growth factors that can interact with pathogen-associated molecular pattern. Moreover, since mitochondria represent a major target of autophagy, deregulated mitochondria autophagy may be involved in the autoimmune pathogenesis occurring in PBC [95]. Finally, in primary sclerosing cholangitis, autophagy and senescence have been associated with the occurrence of epithelial to mesenchymal transition traits in cholangiocytes and biliary tree stem cells, with dysplasia features [96]. Interestingly in the last years, a novel mechanism implicating GSK-3*β* in TGF-*β*-induced EMT program has been reported [97]. Given the role of GSK-3 in regulating autophagy and its role in promoting metabolic changes toward the anabolism, GSK-3 may be considered as a potential target to counteract liver injury associated with autophagy impairment and senescence processes also in biliary tree disorders.

## 6. Role of GSK-3 in Oxidative Stress and Autophagic Cell Death

Oxidative stress occurs when the balance between reactive oxygen species (ROS) production and elimination is altered leading to accumulation of ROS which profoundly affects lipids, proteins, and DNA. Mitochondria are both great producers and main targets of ROS; therefore, they play a central role in oxidative homeostasis. As a consequence of oxidative damage, mitochondrial permeability transition (MPT), a nonselective permeabilization of mitochondria inner membrane, may occur, usually followed by necrotic or apoptotic cell death [98]. GSK-3 activity is induced by ROS and it is involved in MPT. More in detail, GSK-3 is able to direct MPT through the phosphorylation of different targets and GSK-3 inhibition is known to protect from MPT [99]. In addition, GSK-3*β* inhibition has been shown to be required for the stability of Nrf2 transcription factor, a key regulator of the cellular defense against oxidative stress [100].

The prooxidative involvement of GSK-3 overexpression may explain at least in part its role in the pathogenesis of several disorders including cancer [99] as well as many neurological disorders including bipolar disorder [101] and AD [102].

Oxidative stress is often associated with different types of liver injury and plays an important role in the mechanism of acute liver failure (ALF) [103]. The effects of oxidative stress are balanced by antioxidant activities with a variety of enzyme and nonenzyme-mediated mechanisms. Active oxygen-scavenging systems include enzymes such as superoxide dismutase (SOD), glutathione peroxidase (GSH-PX), and catalase, while nonenzymatic antioxidants include GSH, vitamin C, and vitamin E [104]. SOD and GSH activity in ALF is significantly lower as compared to normal controls. The oxidation status enhances paralleling ALF progression, whereas the antioxidants are reduced, resulting in a severe oxidative stress in ALF and in the progression of liver injury [85]. Oxidative stress regulates hepatocyte injury and death, and GSK-3*β* appears to be critical for their regulation in ALF. GSK-3*β* activity is depressed at an early stage of ALF and then goes back to high levels in the advanced ALF, further suggesting that GSK-3*β* may have a role in ALF progression. In hepatic ischemia/reperfusion (I/R) injury, that is, the most common cause of acute hepatic failure (after liver transplantation, hepatectomy, trauma, and shock), reperfusion following prolonged ischemia is related to the mitochondrial dysfunction, which induces liver apoptosis [105]. The impairment of oxidative phosphorylation and induction of MPT are critical determinants for such mitochondrial dysfunction [106] and are dependent on GSK-3*β* activity [107]. It has also been demonstrated that propofol, a drug used to induce and maintain anesthesia, may protect several tissues from I/R injury [108] supporting their mitochondrial function, thanks to GSK-3*β* inhibition which restrains MPT, preventing the cytochrome C release, mitochondrial swell, and mitochondrial membrane potential collapse [105].

GSK-3 has been also reported to play a role in regulating autophagic cell death. Under such condition, extensive autophagy does not provide cytoprotection but triggers cell death. Overexpression of Aurora-A kinase, a serine/threonine protein kinase, enhances mTORC1 activity by antagonizing GSK-3*β* activity, thus conferring resistance to autophagic cell death [109]. Furthermore, in a model of mesangial cells, cadmium has been demonstrated to be able to induce autophagic cell death through a GSK-3-regulated signal-transduction pathway. Serine 9 phosphorylation (i.e., the phosphorylation leading to the GSK-3*β* inhibition) decreases after cadmium treatment and, in turn, a specific GSK-3*β* inhibitor decreases cadmium-induced autophagic cell death. Remarkably, GSK-3 activation after cadmium treatment is a consequence of ROS elevation and a ROS scavenger is able to counteract autophagic cell death [110, 111]. Conversely, activation of AKT and GSK-3*β* inhibition suppresses cytodestructive autophagy in hippocampal neurons [112]. Furthermore, in neural stem cells, following insulin withdrawal, both pharmacological and genetic inactivation of GSK-3*β* significantly decreases autophagic cell death [113]. In addition, GSK-3*β*-mediated phosphorylation of BCL2 family member MCL1 has been demonstrated to induce axonal autophagy and axonal degeneration [114]. Altogether, these data indicate that besides controlling oxidative stress cellular response, GSK-3 may be also involved in prodeath autophagy.

## 7. GSK-3*β*-Specific Inhibitors: Using GSK-3 as a Pharmacological Target

Modulation of GSK-3 activity via pharmacological intervention may represent a valuable strategy to control autophagy and other conditions. In fact, GSK-3 is emerging as a possible therapeutic target for many diseases, and selective GSK-3 inhibitors are now available. Numerous studies show that GSK3 action supports cancer cells and suggest that its inhibition may have therapeutic benefits. However, as highlighted above, GSK-3 role in tumor development is still controversial. Many GSK-3 inhibitors have been developed and may have an application in GSK-3 overexpressing tumors [115]. The cation lithium is the first inhibitor to be discovered. Other metal anions such as copper, beryllium, mercury, and zinc have also been shown to interfere with GSK-3 activity. Other known GSK-3 inhibitors are chemical compounds including natural substances as well as synthetic ATP-competitive inhibitors, non-ATP-competitive inhibitors, and substrate-competitive inhibitors [66, 116]. An issue regarding ATP-competitive inhibitors may concern their lack of specificity; namely, they interfere with the phosphorylation of many substrates, giving potential oncogenic effects [3]. Some GSK-3 inhibitors have been used in clinical trials and are well-tolerated in cancer patients [117]. Significant clinical improvements have been shown in cutaneous T-cell lymphoma patients treated with valproate, which inhibits either GSK-3 isoforms [118].

Different therapeutic strategies to treat leukemia have been shown to involve mechanisms leading to GSK-3 activation often by suppressing PI3K/AKT pathway. For instance, a specific AKT inhibitor induces apoptosis in T-cell acute lymphoblastic leukemia (ALL) through a mechanism partially dependent on GSK-3 activation [119]. A GSK-3 inhibitor named PDA-66 shows some promise in preclinical studies using ALL cells [120] while GS-87, a highly specific inhibitor of GSK3, has been shown to induce differentiation of AML cells [121]. Nevertheless, the potential differentiating effect of GSK3 inhibitors needs to be further explored. The selective GSK-3*α* and GSK-3*β* inhibitor LY2090314 shows very high cytotoxic activity in melanoma cells, both resistant and nonresistant to BRAF inhibitor. Such activity was strongly associated with *β*-catenin stabilization. *In vivo* confirmation of such data further support the potential efficacy of GSK-3 inhibitors in melanoma [122].

While different GSK-3 inhibitors have been evaluated in several pathologies and are well-tolerated in leukemia and pancreatic cancer patients, no clinical trials have been performed or are currently ongoing in HCC patients. Only preclinical studies are available on GSK-3 inhibitors in HCC. Indeed, developing novel GSK-3 inhibitors might be crucial to identify novel GSK-3 substrates and novel GSK-3 functions specific for one of the two isoforms. GSK-3 inhibitors have been tested in neurodegenerative conditions [123]. Unfortunately, sodium valproate [124] and tideglusib (a non-ATP competitive GSK-3 inhibitor) have both shown no significant effect in progressive supranuclear palsy [125, 126] while contrasting results are raised for tideglusib-treated Alzheimer's disease patients in two different clinical trials [127, 128]. Nevertheless, significant clinical improvements have been shown in valproate-treated patients affected by chronic migraine [129].

## 8. Does GSK-3 Counteract Mammalian Aging?

The GSK-3 ability to regulate numerous cellular processes through a number of signaling pathways important for cell proliferation, stem cell renewal, apoptosis, and development is widely accepted [130]. Because of its multifunctional role, GSK-3 strongly affects the first stages of human diseases as well as regulates age-related pathologies. Four main theories underlying aging molecular process are now generally accepted. Three of them are based on telomere loss, somatic mutation, and mitochondrial action. These hypotheses take into account, respectively, telomere shortening dysfunction, forms of DNA damage exciding DNA repair capacity, and mutation of mitochondrial DNA impairing ATP production. The fourth theory regards the waste accumulation, that is, it hypothesizes the aging results from toxic protein accumulation and alteration of degradative mechanisms such as lysosome-mediated autophagy [131, 132].

Metabolic alterations such as mitochondrial dysfunction, as previously mentioned, characterize senescent cells displaying structural features such as enlarged volume, increased granularity, and oxidative stress, all falling under GSK-3 control. Kim and colleagues [133] demonstrated that different anabolic processes, such as lipogenesis, glycogenesis, and protein synthesis increase during senescence in primary cell cultures. Consequently, the mass of senescent cells is augmented. Such increase is accompanied by ROS overproduction caused by defective respiration [134]. Oxidative stress induces and maintains the senescence cellular phenotypes since mitochondrial DNA is susceptible to oxidative damage. GSK-3 inactivation through phosphorylation plays a key role in these aging processes; in fact, GSK-3 is directly involved in glycogen accumulation as well as in protein synthesis activation, characterizing senescent cells [135]. The correlation between mitochondrial defects and metabolic changes related to age as well as the link with GSK-3 has been demonstrated by Kim and colleagues [133] using immortalized human liver cell, Chang cells, exposed to deferoxamine to induce senescence. Deferoxamine augmented GSK-3 phosphorylation at both serine 9 of GSK-3*β* and serine 21 of GSK-3*α* causing strong glycogen accumulation. Remarkably, the increase of the intracellular organelles like lysosomes and mitochondria [136], endoplasmic reticulum, and Golgi as well as total cell lipid content, represents a defense response to oxidative stress and a senescence factor. Namely, SREBP1 transcription factor expression, the major modulator of lipogenic enzyme modulator [137], is a GSK-3 target and increases in cell senescent systems. According to this finding, Kim and colleagues [133] observed that GSK-3 inhibition augmented cellular lipogenesis and membranous organelle mass. Grune and colleagues described an increase in the nonmembranous organelles [138]. This effect is related to a higher cellular anabolism during senescence, when cells are exposed to persistent oxidative stress with potential damage of cellular organelles.

Hence, GSK-3 inhibition leads to intracellular ROS overproduction thus stimulating mitochondrial damage. Furthermore, GSK-3 controls master factors in anabolic activation (namely, eIF2B, glycogen synthase, and SREBP1). Therefore, GSK-3 can be considered a main factor of the metabolic changes towards the anabolism shift observed in senescence.

Interestingly, some medicinal plants display antiaging effects shown to be linked to GSK-3 pathway regulation. In particular, several natural or nutraceutical products are suggested to have health-ameliorating effects or antiaging and anticancer effects. Such effects are modulated by PI3K/PTEN/AKT/mTORC1/GSK-3 signaling axis. Namely, three medicinal plant-derived substances are involved in the above-cited regulation: curcumin (CUR) *Curcuma longa*, berberine (BBR) Berberiscoptes, and resveratrol (RES), the latter especially present in red grapes. CUR acts by increasing the total level of GSK-3*β* in NCCIT human embryonic carcinoma cells with apoptosis induction, and a plethora of studies in the last years underlined the favorable impact of CUR on PI3K/PTEN/AKT/mTORC1/GSK-3 pathway in different types of cancer [139] and pathologies such as neurological diseases [140], obesity [141], diabetes [142], and cardiovascular disease [143]. BBR and RES act on PI3K/PTEN/AKT/mTORC1/GSK-3 pathway with beneficial effects on diabetes, cardiovascular diseases, neurological disorders, and cancer [144].

In conclusion, dietary or pharmacological administration of these compounds may represent, at least to some extent, potential alternatives to conventional drugs and still underlies the efficacy of GSK-3 modulation in counteracting aging-related pathologies.

## 9. Conclusions

Altogether, the studies summarized in the present review show that GSK-3 controls numerous cellular processes, plays an important role in autophagy regulation, and is involved in many human diseases. Further investigating substrate specificity and regulation of GSK-3 activity has important implications for potential therapeutic intervention.
